# The Indications for Ventral Suspension of the Uterus

**Published:** 1897-02

**Authors:** 


### THE INDICATIONS FOR VENTRAL SUSPENSION OF THE UTERUS.

Dr. Augustin H. Goelet, of New York, at the recent meeting of the Mississippi Valley Medical Association (*International Journal of Surgery*) presented a paper on this subject. He drew especial attention to the difference between ventral suspension and ordinary ventro-fixation. The latter fixes the uterus permanently by adhesion against the anterior abnominal wall and substitutes another abnormal position which may be a little better than the original displacement. Ventral suspension attaches the uterus to the anterior abdominal wall by two sutures only, which include only the peritoneum and subperitoneal fascia of the abdominal wall and the few muscular fibres of the posterior face of the fundus of the uterus. At first the uterus is drawn up close against the abdominal wall, but subsequently recedes, the attached surfaces stretching out and forming a strong fibrous band of about an inch or an inch and a half in length. The uterus is, therefore, suspended from the anterior abdominal wall in a nearly normal ante-position and it is fairly movable.

The special indications for this operation are considered to be retro-deviation of the uterus fixed by adhesions and prolapse of the uterus. The author believes, also, that the uterus should be thus suspended whenever both appendages have been removed, and in consequence it has been deprived of the support of the broad ligaments.

